# Extending Methods in Dietary Patterns Research

**DOI:** 10.3390/nu10050571

**Published:** 2018-05-07

**Authors:** Jill Reedy, Amy F. Subar, Stephanie M. George, Susan M. Krebs-Smith

**Affiliations:** 1 Rick Factor Assessment Branch, Epidemiology and Genomics Research Program, National Cancer Institute, 9609 Medical Center Drive, Bethesda, MD 20892, USA; subara@mail.nih.gov (A.F.S.); krebssms@mail.nih.gov (S.M.K.-S.); 2 Office of Disease Prevention, Office of the Director, National Institutes of Health, 6100 Executive Blvd, Rockville, MD 20852, USA; stephanie.george@nih.gov

**Keywords:** dietary patterns, diet assessment, nutritional epidemiology, multidimensionality, dynamism, life course

## Abstract

The National Cancer Institute (NCI) and the National Institutes of Health (NIH) Office of Disease Prevention held a workshop titled, “Extending Methods in Dietary Patterns Research”, in May of 2016. The workshop’s goal was to articulate, refine, and prioritize methodological questions to advance the science of dietary patterns in epidemiological research. Although the focus was on how to improve methods for assessing the relationship between dietary patterns and cancer risk, many, if not all, of the discussions and conclusions are relevant for other health outcomes as well. Recognizing that dietary intake is both multidimensional (i.e., it is a complex, multi-layered exposure and behavior) and dynamic (i.e., it varies over time and the life course), workshop presenters and participants discussed methodological advances required to include these concepts in dietary patterns research. This commentary highlights key needs that were identified to extend methods in dietary patterns research by integrating multidimensionality and dynamism into how dietary patterns are measured and defined, and how relationships with dietary patterns and health outcomes are modeled.

## 1. Introduction

Most of the evidence base for diet and cancer prevention has emerged from analyses focused on single nutrients and food groups. This reductionist, or decompositional, approach—emphasizing specific dietary constituents rather than dietary patterns—aligns with early research that helped to define the field of nutrition science through landmark discoveries related to nutrient deficiencies. Within a cancer model, such an approach assumes that a discrete dietary chemical has a specific biological effect on carcinogenesis [1,2]. However, this approach presents several limitations and challenges because nutrients and foods are not typically consumed in isolation. First, intakes of many individual dietary constituents are highly correlated, making it difficult to examine the real association between any one and a cancer outcome. Second, the totality of diet likely has interactive, synergistic, and combined effects, so such a “precisely focused approach may overlook the significance of diets as a whole [3]”. Thus, a holistic, or integrative, approach—considering the totality of diet began to evolve. This complementary approach assumes that there is biochemical complexity; the multitude of dietary nutrients and constituents (some measured, and some perhaps unknown) are correlated and interrelated biologically with the potential to influence the initiation and development of cancer [2,4,5].

Examining the totality of the diet, and considering multiple components simultaneously as a dietary pattern, has become a more recent focus of diet and cancer research. But progress is hampered by the existence of multiple methods used to define a pattern and lack of standardization within methods. Dietary patterns are conceptualized and defined in many ways: as an exposure or a behavior, by numbers or labels, as univariate or multivariate constructs, as research-driven or data-driven, and as static or dynamic. Comprehensive reports such as the 2007 World Cancer Research Fund/American Institute for Cancer Research Expert Report have stated that the literature in this area is too heterogeneous to draw firm conclusions on the role of diet patterns in cancer [3]. Similarly, although dietary patterns were at the core of the 2015 Scientific Report of the Dietary Guidelines Advisory Committee [6] the Committee noted that the disparate nature of the dietary patterns methods used made it challenging to compare results across studies, and for some methods and outcomes, few studies were available for comparisons. Their report also highlighted recommendations for future research, which included the need to “understand better the concept of dietary patterns and design approaches to quantify diet in large population-based studies [6].”

## 2. A Dietary Patterns Methods Workshop

Given these reports and recommendations, the National Cancer Institute (NCI) and the National Institutes of Health (NIH) Office of Disease Prevention held a workshop titled, “Extending Methods in Dietary Patterns Research”, in May of 2016. The workshop’s goal was to articulate, refine, and prioritize methodological questions to advance the science of dietary patterns in epidemiological research. Although the focus was on how to improve methods for assessing the relationship between dietary patterns and cancer risk, many, if not all, of the discussions and conclusions are relevant for other health outcomes as well.

The workshop brought together more than 50 nutritionists, epidemiologists, and statisticians to discuss dietary patterns methods research, with participants coming from 23 academic institutions representing seven countries (United States, Canada, United Kingdom, France, Netherlands, Germany, and Israel), as well as from two international cancer organizations (International Association for Cancer Research and World Cancer Research Fund), six institutes and offices at the National Institutes of Health (including the National Cancer Institute; National Heart, Blood, and Lung Institute; National Institute for Digestive and Kidney Diseases; Eunice Kennedy Shriver National Institute of Child Health and Human Development; and the National Institute for Alcohol Abuse and Alcoholism; and the Office of Disease Prevention), and two other federal agencies (United States Department of Agriculture and the Food and Drug Administration).

## 3. Integrating Multidimensionality and Dynamism in Dietary Patterns

This commentary focuses on the existing gaps, identified in the workshop, for fully integrating multidimensionality and dynamism into epidemiologic research models to better inform the understanding of the relationship between dietary patterns and cancer prevention and progression.

### 3.1. Dietary Patterns

The workshop’s starting point was the traditional definition of dietary patterns, which is “the quantities, proportions, variety, or combination of different foods, drinks and nutrients in diets, and the frequency with which they are habitually consumed [7].” However, recognizing that dietary intake is both multidimensional (i.e., it is a complex, multi-layered exposure and behavior) and dynamic (i.e., it varies over time and life course developmental stage), workshop presenters and participants discussed methodological advances required to include these concepts in dietary patterns research.

### 3.2. Multidimensionality

Attempts to examine the totality of diet have customarily used a summary index that includes all aspects of the diet on the same scale. More recently, there has been an appreciation that the different dimensions of diet act differently and therefore should be considered separately but collectively. Some dimensions are to be enhanced, and some are to be constrained. Some dimensions have strong correlations with other aspects of diet, while others may have inverse, converse, or no correlations at all. Additionally, epidemiological data suggest that there are some dimensions, such as alcohol, that has both positive and negative effects depending on the outcome under investigation (cardiovascular disease, breast cancer). Multidimensionality refers to the numerous attributes of dietary intake and the inherent complexities of interdependence and synergy. For example, dimensions may be based on vitamins, minerals, macronutrients, botanical grouping systems, guidance-based foods groups, categories of foods as-eaten, chemical constituents (such as types of polyphenols), contaminants, and others. As noted above, there have been wide-ranging and varying approaches regarding which dietary dimensions best reflect a dietary patterns construct and might be most relevant for a given research question.

### 3.3. Dynamism

The construct of diet is typically based on a single assessment and assumes that diet at a single period of time is the exposure of interest, or that diet is unchanging. Yet for most people, diet is dynamic and time-varying—changing over days of the week, weeks of the year, and throughout the lifecycle. Relevant life stages may include critical developmental periods (such as preconception, conception, in utero, birth, childhood, adolescence, pregnancy, adulthood, and older adulthood), or at different phases in the cancer continuum (such as prevention, treatment, and survivorship). Additionally, dynamism has relevance in the shorter-term, with questions related to how meal frequency, intervals between eating occasions, and timing of food combinations effect metabolism, circadian rhythm, and health.

## 4. Research Gaps

To further advance the science of dietary patterns in epidemiological research, ten research gaps, with a summary of their background, current status, and goals for next steps, were identified:

1. Need for a shared conceptual framework
  - Issue: There is inconsistency in the language and conceptual framework for dietary patterns in epidemiology.
  - Status: Tensions exist regarding how to concomitantly characterize the effects of individual dietary constituents, and of the total diet, or dietary pattern.
  - Goal: Clarifying a conceptual framework for dietary patterns in nutritional epidemiology would better enable the synthesis of studies with dietary patterns and improve the translation of findings for policy, guidance, and intervention [8]. Additionally, there is a need for this framework to further extend discussions and knowledge regarding the relationships between intake and multiple biological response indicators (e.g., biologic levels of nutrients, or markers of cell function/dysfunction). Such efforts would facilitate transparency in the development of dietary guidelines and help refine how to best identify and characterize different dimensions of exposure and relevant time periods.
2. Need to develop improved diet assessment tools to include contextual and dynamic attributes of dietary patterns
  - Issue: Considering that dietary exposures and health conditions likely exert influence over time—through diurnal variations or, over the long term, cumulatively or at critical periods of development—the ability to time stamp eating occasions throughout the day and capture data over the life course would provide tremendous opportunities to explore these areas more effectively.
  - Status: Tools such as food frequency questionnaires (FFQs) have been used at repeated time points to assess the effect of change and the potential for change [9]; however, FFQs do not capture short-term within person variation to allow for investigation of diurnal variation or timing, and the inherent structure cannot allow for examination of combinations of foods consumed together in meals. Other existing tools such as 24-hour recalls and records can provide information related to a shorter time-frame such as hourly variation and meal patterns, and also over a longer time-frame through repeated measurements.
  - Goal: New technologies and improved tools, including cameras, microphones, and pattern recognition, would allow integrating information on what we eat and how we eat it, including meal patterns, timing, and other contextual attributes. Advanced methods of data capture, storage, and retrieval may help obtain the requisite data over time that to address aspects of dynamism. Lastly, questions exist regarding the feasibility for integrating emerging biomarkers and other technologies to examine potential metabolomics profiles or microbiome signatures for different dietary patterns.
3. Need to standardize dietary patterns methods and scores
  - Issue: Within a shared conceptual framework, standardized guidelines, centralized data collection, and data repositories that are readily available to the larger research community could allow for pooling and other coordinated analyses. However, differences in diet assessment, databases, and methods have made it challenging to compare and synthesize across cohorts, including seemingly simple issues such as translating cups, servings, and grams.
  - Status: A total diet quality score is unidimensional, but it is built up from a vector of scores with different values. Standardization of input variables and algorithms, and harmonization of methods is required, as is nuance in interpreting and capturing the degree of concordance with a pattern.
  - Goal: Some efforts such as the Dietary Patterns Methods Project that are already underway could be further extended to standardize methods and scores used, and apply different scores in populations for within and between country comparisons [10].
4. Need to develop methods and models that fully capture the richness within the total dietary pattern
  - Issue: Statistical methods are needed to allow for models that use more or all of the multidimensional attributes of the diet quality index concept to identify and describe dietary patterns, evaluate changes, and examine what patterns most explain health outcomes.
  - Status: Several diet quality indices have been conceptualized as constructs that incorporate multiple dimensions of diet simultaneously (e.g., multiple food groups), yet this information is then typically reduced to a single, unidimensional diet quality metric or score (as in the Mediterranean Diet Score, or Healthy Eating Index). Although a summary score can be instructive when scores are high (or low), such a unidimensional score may not allow for appropriate differentiation to understand how different patterns of eating may lead to a similarly high score. Scores in the middle include individuals with the same score, but diets that may vary based on different dimensions. Efforts are underway that attempt to untangle the multiple dimensions with data visualization techniques and statistical approaches [11,12].
  - Goal: An emphasis is needed on models that account for the totality of diet, with the whole as more than a sum of its parts, that can discern the differing diet profiles, or the multiple ways to achieve a healthy dietary pattern.
5. Need to clarify the appropriate methods and models to interpret substitution effects for single components within the context of a total dietary pattern
  - Issue: Increasing or decreasing consumption of a single aspect of diet, is typically connected to other aspects of intake. Making a conclusion about the impact of the presence or absence of food X, or sugar-sweetened beverages, red meat, or alcohol, within the context of the total dietary pattern, would be powerful.
  - Status: The Dietary Guidelines Report recommends several healthy eating patterns including the Healthy US-style Pattern, the Healthy Mediterranean-style Pattern, and the Healthy Vegetarian Pattern [6], but consumers want to know which one is best, and whether the optimal patterns and trade-offs among foods are the same for everyone.
  - Goal: Understanding substitution issues, or how specific foods matter in the context of different dietary patterns, is critical for recommendations and interventions. There is a need to further understand whether different patterns (or variants of patterns) of overall high quality are equally protective for all populations.
6. Need to develop patterning tools based on both etiology (across cancer continuum) and prediction (to optimize biomarkers, outcomes)
  - Issue: Linked to the tension between a single dietary constituent and total dietary patterns, is the tension between etiology and prediction. Concerns exist that the emergence of dietary patterns means a move away from etiology.
  - Status: Diet quality indices are increasingly popular because they are comparable across diverse study populations. The risk prediction ability is enhanced, but the findings regarding etiology and ability to conceptualize whole body metabolism is somewhat blurred.
  - Goal: Although some questions may be grounded in etiology (for scientific understanding) and others in prediction (to inform health policy, dietary guidelines, and interventions), they are both relevant [13]. Supervised learning might be used to form clusters, factors, or other groupings to understand the multidimensional nature of diet, but in such a way that the clusters are informed by health outcomes. Model-building might occur from the ground-up, or from the top down [14].
7. Need to consider timeframes or relevant periods of timing, apply time-varying models for dietary patterns across the life course
  - Issue: Dietary patterns and food choices change over the course of a lifetime, sometimes in dramatic and long-lasting ways. When these changes occur, and how they interact with the biological, developmental, or sociocultural context in which they occur, may influence disease risk in profound ways.
  - Status: Attempts to apply these realizations have begun in the area of breast cancer etiology [15]; and there are other efforts with time varying models with other exposures and conditions [16,17]. However, applying insights regarding the life course and its influence on lifestyle factors to studies of diet, dietary patterns, and cancer are just beginning [18].
  - Goal: The development of a “synthetic” cohort could be created to link the effects of dietary patterns and outcomes in one stage of life to parallel studies in another stage. Such an approach would facilitate an understanding about which periods of exposure are most important in cancer risk, and by defining dietary patterns trajectories may also allow opportunities for intervention and anticipation of possible transition points.
8. Need to consider timing and frequency of dietary patterns over the short term such as by meal, by day
  - Issue: There are granular questions regarding the acute effects of meals, food combinations, meal timing, and other behavioral aspects of intakes on metabolism, hormone secretion, circadian rhythm, and other biological response indicators. There have been challenges summarizing the existing literature due to variations in definitions, and lack of data in large cohorts that capture intake such that timing and combinations of foods can be considered.
  - Status: A few studies have shown biological consequences regarding the number of meals or eating occasions per day, meal skipping, hours between eating occasions, and/or fasting [19,20].
  - Goal: Develop models able to examine the health effects over time for short-term eating behaviors and dietary patterns to clarify appropriate dietary recommendations across the cancer continuum and throughout phases of treatment.
9. Need to evaluate the effect of measurement error in dietary patterns and develop methods to adjust for this error
  - Issue: Given the complexity of dietary patterns, adjusting for measurement error may or may not be viable. However, measurement error correction is likely necessary, so it is critical to understand the impact of the problem.
  - Status: The extent and effect of measurement error in diet has been well documented [21,22]. However, understanding how measurement error affects hazard ratios for major dietary indices and other dietary patterns is not yet well-articulated in the literature.
  - Goal: Sensitivity analyses should be pursued to lead to better understanding of the effect of measurement error in these models. There is a need to develop flexible methods to characterize trajectories of usual intake and to appropriately adjust for measurement error in models with health outcomes.
10. Need to include more systems-oriented approaches, that consider measures of other related exposures and their interactions within the context of dietary patterns.
  - Issue: Multidimensionality and dynamism play roles for all exposures and outcomes. Models need to include dietary patterns within the context of other exposures, and varying outcomes including cancer, cardiovascular disease, mortality, composite outcomes, quality-adjusted life years, and healthy aging.
  - Status: The fields of dietary patterns research and complex systems modeling have yet to converge in a substantive way [23,24].
  - Goal: There is a need to orient data collection and other aspects of studies to incorporate systems approaches and models. Study designs and analysis methods can consider how to include other lifestyle variables and psychosocial factors that influence dietary patterns.

## 5. Summary

A focused emphasis on the concept of dietary patterns and study design to measure dietary patterns in large cohorts can advance nutritional epidemiology. Additionally, efforts to build capacity among nutritionists, statisticians, and epidemiologists will further support broader application of innovative methods to address multidimensionality and dynamism. To best understand the role of dietary patterns, it is important to consider how diet is measured, defined, and modeled because each of these influences the ability to examine and interpret dietary patterns and cancer relationships. The needs identified and discussed during this workshop should help to stimulate more research to integrate multidimensionality and dynamism into dietary patterns methods research, and enable well-defined methods and models to examine the potential associations between the totality of diet over the life course and cancer prevention and control.
